# A novel fast strategy to calculate equieffective doses under different dose rate conditions

**DOI:** 10.1002/mp.17688

**Published:** 2025-02-15

**Authors:** Mark J. Macsuka, Roger W. Howell, Katherine A. Vallis, Daniel R. McGowan

**Affiliations:** ^1^ Department of Oncology Radiobiology Research Institute Churchill Hospital University of Oxford Oxford UK; ^2^ Department of Radiology Rutgers New Jersey Medical School New Jersey USA; ^3^ Department of Medical Physics and Clinical Engineering Churchill Hospital Oxford University Hospitals NHS Foundation Trust Oxford UK

**Keywords:** biologically effective dose, equieffective dose, radiopharmaceutical therapy

## Abstract

**Background:**

Radiopharmaceutical therapy (RPT) has gained notable attention for its potential in treating difficult cancers, with [^177^Lu]Lu‐DOTATATE being a notable example. However, the radiobiology of RPT is less understood compared to external beam radiotherapy (EBRT), and dosimetry protocols are not standardized. Organ dose limits and tumor dose‐response correlations are often based on radiobiologically motivated equieffective doses (EQDX). On top of absorbed dose, these measures are also functions of the absorbed dose rate and radiobiological parameters that quantify tissue radiosensitivity and damage repair rate. Typically, the absorbed dose and repair rates are assumed to follow a monoexponential pattern, although describing the dose rate function often requires two or more phases to describe the data.

**Purpose:**

Here we present novel expressions for calculating the equieffective dose in 2 Gy fractions (EQD2) for RPT, considering various absorbed dose rate scenarios and the rate of sublethal DNA damage repair. We aimed to establish an approach that is scalable, robust, and can be used alongside various absorbed dose integration methods.

**Methods:**

By assuming a simple exponential decay for DNA damage repair and employing a biexponential function for absorbed dose rate decay, we have re‐established the solutions for EQDX in a concise analytical form. Additionally, we have devised a novel hybrid solution applicable to piecewise‐defined absorbed dose‐rate functions, leveraging both numerical and analytical methodologies. To validate these expressions, simulated measurements were utilized, and comparisons were made with a fully numerical approach. We also investigated the reliability of three methodologies—fully numerical, fully analytical, and a hybrid approach—when simplifying comprehensive dosimetry protocols. Utilizing publicly available clinical data from two patients undergoing [^177^Lu]Lu‐DOTATATE therapy, we defined the baseline absorbed dose rate model based on the best biexponential fit to four post‐injection SPECT measurements at the organ level. We then explored variations in EQD2 values resulting from the omission of the final measurement.

**Results:**

The proposed expressions were found to be accurate and scalable, providing a reliable alternative to fully numerical methods. The results of the fully numerical method converged to our solutions with increasing accuracy as the extrapolation time after injection was increased. However, we found that to achieve an accuracy in EQD2 to within 2%, the numerical method had to extrapolate for up to 890 h in some cases, at which point overflow errors are likely to occur. Our hybrid method also achieved a significant decrease in computation time compared to the fully numerical method.Using data from two patients, we found that the numerical, hybrid, and analytical approaches underestimated the baseline EQD2 to tumors by 15.6 ± 9.4 %, 5.0 ± 4.2 %, and 1.5 ± 2.9 %, respectively.

**Conclusions:**

Comprehensive dosimetric studies are often preferred in RPT when increased measurement accuracy is desired. Correspondingly, it is vital for radiobiological models to maintain a level of accuracy commensurate with comprehensive studies. Our proposed methods are accurate, scalable, and suitable for radiobiologically motivated RPT dosimetry.

**Abbreviations**
$\dot{D}$
: absorbed dose rate
$T_{rep}$
: DNA repair half‐time
$r_{0}$
: biexponential absorbed dose rate coefficient
$t_{x}$
: time of measurement number x
$\lambda_{e_{1}},\lambda_{e_{2}}$
: effective decay constants
$\lambda_{1},\lambda_{2}$
: biological clearance parameters
BED: biologically effective dose
d: absorbed dose per fraction
D: total absorbed dose
EBRT: external Beam Radiotherapy
EQDX: equieffective dose in X Gy fractions
G: Lea‐Catcheside factor
N, M: numerical integration results in the hybrid approach
RE: relative effectiveness
RPT: radiopharmaceutical therapy
α, β: radiosensitivity parameters
λ
: physical decay constant
μ
: DNA repair constant
ψ
: DNA repair model

## 1 INTRODUCTION

Radiopharmaceutical therapy (RPT) is a therapeutic modality that has gathered a substantial amount of interest in the past few years, with numerous clinical trials showing great promise in the control of various hard‐to‐treat cancers.^1^ The results of the NETTER‐1 trial established RPT and specifically [^177^Lu]Lu‐DOTATATE as a viable treatment option for patients with neuroendocrine tumors.^2^ The subsequent FDA approval invigorated research in the field of RPT with ^177^Lu and various other *α‐* and *β‐*emitting radionuclides labeled to a range of peptides and antibodies^1^. The recent FDA approval of ^177^Lu‐labeled PSMA‐617 for the treatment of metastatic castration‐resistant prostate cancer is expected to further drive research into the optimization of current and novel RPTs.^3^

In contrast to external beam radiotherapy (EBRT), however, the radiobiology of RPT is poorly understood and dosimetry protocols are not optimized, let alone standardized across different treatment centers.^4^, ^5^, ^6^ Even though guidelines do exist, they only pertain to optimizing the accuracy of dosimetry. For example, ICRU Report 96 calls for at least four scans following administration of [^177^Lu]Lu‐DOTATATE for the ideal modeling of tumor tissue kinetics.^7^ However, this is seldom followed in daily clinical practice because it requires patients to reattend for multiple scans and pressure to constrain costs. There is an extensive and growing literature discussing simplified dosimetry protocols and absorbed dose calculation methods,^8^, ^9^, ^10^ but the same cannot be said for radiobiological models. It is not obvious that the same simplifications would remain optimal or even appropriate when using more advanced dose‐related radiobiological metrics, such as the biologically effective dose (BED) or the equieffective dose (EQD*X*), which are well‐established concepts used to compare and combine the effects of different treatment schedules and modalities. Additionally, how exactly the absorbed dose is calculated is not standardized between sites and dosimetry software vendors and it depends on which time points are considered.^7^, ^11^, ^12^ Therefore, it is highly warranted that such metrics are calculated accurately before the effects of using a reduced number of measurements are considered.

An expression for the EQD*X* can be derived from the consideration of clonogenic cell survival using the linear‐quadratic (LQ) model. In general, with respect to EBRT given in *X* Gy fractions, it takes on the following form^13^:

(1) $$\;\mathrm{EQD}X_{\alpha /\beta }=\;D\frac{1+\frac{d}{\alpha /\beta }}{1+\frac{X}{\alpha /\beta }}$$

where *D* is the total absorbed dose, *d* is the absorbed dose per fraction of the test therapy, and α/β quantifies tissue radiosensitivity.

The BED can be written as a special case of Equation 1 with vanishing reference doses per fraction:

(2) $$\mathrm{BE}D_{\alpha /\beta }\equiv \mathrm{EQD}\;0_{\alpha /\beta }=\;D\cdot \mathrm{RE},\;$$

where the mathematical form of the relative effectiveness, RE, depends on the details of irradiation and the DNA damage repair model used. For EBRT, the RE is the numerator of the ratio presented in Equation 1.

During RPT with β‐emitters, irradiation happens continuously and simultaneously with DNA damage repair. Therefore, a dependence on the absorbed dose rate and a model of DNA damage repair must be included in RE, usually through the addition of a dose protraction (or G) factor, which is an explicit function of absorbed dose rate and depends on the sublethal damage repair model used.^14^ It can be shown that:

(3) $$\mathrm{RE}\;=1+\frac{G\left(\dot{D}\left(t\right),\;\psi \left(t\right)\right)}{\alpha /\beta }\cdot D$$

where the *G* factor is an explicit function of absorbed dose rate $\dot{D}\left(t\right)$ and a time‐dependent probabilistic repair model ψ(t). *G* can be interpreted mechanistically as the ratio of total non‐immediate lethal events with (*R*) and without (*R*_0_) repair. The explicit mathematical form of *G* was first motivated by modeling the repair of chromosome breaks after continuous irradiation until a time τ, but it is worth pointing out that it can be derived from other mechanistic models and is quite general in that sense.^14^, ^15^ Assuming complete decay and a single‐phase exponential repair model, it can be written as:

(4) $$\begin{array}{cc} G & =\;\frac{R}{R_{0}}=\frac{\int_{0}^{\tau }dt\;\dot{D}\left(t\right)\int_{0}^{t}dt^{\prime }\dot{D}\left(t^{\prime }\right)\psi \left(t-t^{\prime }\right)}{\int_{0}^{\tau }dt\;\dot{D}\left(t\right)\int_{0}^{t}dt^{\prime }\dot{D}\left(t^{\prime }\right)}\\ & =\frac{\int_{0}^{\infty }dt\;\dot{D}\left(t\right)\int_{0}^{t}dt^{\prime }\dot{D}\left(t^{\prime }\right)e^{-\mu \left(t-t^{\prime }\right)}}{D^{2}/2\;}\;, \end{array}$$

where *t*’ is a dummy variable. The denominator can always be solved analytically, as outlined in the Appendix. The exponential form is the most popular and best‐established choice for the repair function. It is derived from the assumption that the rate of repair is proportional to the number of repairable lesions, which results in a decaying exponential with a characteristic repair time *μ*.^16^ Note that the repair half‐time is defined by $T_{rep}=\ln\left(2\right)\;/\mu$. This parameter can be measured by for example, the *γH*2*AX* or clonogenic assays.^16^, ^17^

Although generalized EQDX calculation frameworks do exist,^18^ for simplicity in the following we assume that both the reference and test radiations trigger the same radiobiological response, such that the α/β parameters are identical. We will compare an analytical, a numerical, and a hybrid approach to calculating the *G* factor. We present two novel expressions for calculating the *G* factor and validate them using simple simulated measurements. First, we derive a simple closed‐form expression for the *G* factor in the case of a biexponential absorbed dose rate function, which is often an appropriate model to describe clinical data.^7^ This first expression is a more compact simplified version of the solution to this problem first presented by Howell et al. in 1994 and 1998.^19^, ^20^ Then, we propose a hybrid numerical‐analytical method for when the previous model is not appropriate and the absorbed dose rate function is piecewise‐defined between measurements, and extrapolation beyond the measurement is assumed to take the form of a mono exponential. The hybrid method is an extension of a fully numerical method first presented by Hobbs and Sgouros in 2009.^21^

After validating our expressions, we further explore the differences between these approaches using a publicly available dataset.^21^ We first establish a baseline model based on the complete dataset made up of four post‐treatment scans and explore how the omission of the final scan affects results, using different calculation methods. We found non‐negligible differences that could lead to significant EQDX underestimation compared to the baseline model, potentially affecting dose‐response inferences.

## 2 METHOD

### 2.1 Methods to calculate the *G* factor

#### 2.1.1 Biexponential absorbed dose rate

ICRU guidelines recommend at least four timepoints for accurate pharmacokinetic modeling in the context of RPT dosimetry, to be able to estimate a biexponential time‐activity curve, which was found to be a good fit to most multi‐timepoint activity measurements of [^177^Lu]Lu‐DOTATATE therapy.^7^ To that end, we will consider that a biexponential function is the standard model of the absorbed dose rate $\dot{D}\left(t\right)$ such that it can be described by

(5) $$\dot{D}\;\left(t\right)=\left(Ae^{-\lambda_{1}t}+Be^{-\lambda_{2}t}\right)\;\cdot e^{-\lambda t}$$

where *A*, *B*, *λ*_1_, and *λ*_2_ are the parameters of the model, and *λ* is the physical decay constant of the radionuclide. Note that the term in parenthesis describes biological clearance while the latter term accounts for the decay of the radionuclide. The assumption that the absorbed dose rate functions are well‐described by biexponential models is expected to hold if they are appropriate models for the time‐activity curves and unless the absorbed dose from cross‐irradiation is highly significant (e.g., ^90^Y), which in the context of data obtained from clinical imaging is unlikely for most RPT radionuclides, which deposit most of their energy in their source voxel (e.g., ^177^Lu). To simplify the above expression, in RPT we can assume that $\dot{D}\left(0\right)=0$, such that *B *= *−A*. Adopting the notation *A *= *r*_0_, Equation 5 simplifies to

(6) $$\dot{D}\;\left(t\right)=r_{0}\;\left(e^{-\lambda_{1}t}-e^{-\lambda_{2}t}\right)\cdot e^{-\lambda t}$$

which can be fitted to successive values of absorbed dose rate. For conciseness, we can redefine the parameters of the biexponential such that

(7) $$\dot{D}\;\left(t\right)=r_{0}\;\left(e^{-\lambda_{e_{1}}t}-e^{-\lambda_{e_{2}}t}\right)$$

where $\lambda_{e_{1}}$ = *λ*_1_ + *λ* and $\lambda_{e_{2}}$ = *λ*_2_ + *λ*. We are now ready to insert this into Equation 4. After integration, the *G* factor will take the form:

(8) $$G=\frac{\lambda_{e_{1}}\lambda_{e_{2}}\left(\lambda_{e_{1}}+\lambda_{e_{2}}+\mu \right)\;}{\left(\lambda_{e_{1}}+\lambda_{e_{2}}\right)\left(\lambda_{e_{1}}+\mu \right)\left(\lambda_{e_{2}}+\mu \right)}$$

and consequently, the RE will be described by:

(9) $$\mathrm{RE}\;=1+\frac{\lambda_{e_{1}}\lambda_{e_{2}}\left(\lambda_{e_{1}}+\lambda_{e_{2}}+\mu \right)\;}{\left(\lambda_{e_{1}}+\lambda_{e_{2}}\right)\left(\lambda_{e_{1}}+\mu \right)\left(\lambda_{e_{2}}+\mu \right)}\;\frac{D}{\alpha /\beta \;},$$

which is a special case of more general expressions first presented in 1994 and 1998.^19^, ^20^ A slightly more involved derivation is in the Appendix. It is also possible to simplify further by using the expanded form of *D*, yielding:

(10) $$\mathrm{RE}\;=1+r_{0}\frac{\left(\lambda_{e_{1}}+\lambda_{e_{2}}+\mu \right)\;\left(\lambda_{e_{2}}-\lambda_{e_{1}}\right)}{\left(\lambda_{e_{1}}+\lambda_{e_{2}}\right)\left(\lambda_{e_{1}}+\mu \right)\left(\lambda_{e_{2}}+\mu \right)\alpha /\beta }\;$$

#### 2.1.2 Piecewise defined absorbed dose rate

In the case when one is not able to adequately fit a biexponential function to the full dataset, or if the number of scans is insufficient to use such a model, it might be advantageous to resort to a fully numerical solution. It has been argued that linear interpolation between data points is an appropriate substitute in some cases, at which point the absorbed dose rate function becomes piecewise linear up until the time of the last one or two scans. Extrapolation beyond that can be achieved by assuming single‐phase decay with a decay constant equal to either the physical decay constant of the radionuclide or the combined physical and biological decay. The latter can be estimated from the last two data points, if appropriate.

Mathematically, the linear parts of the absorbed dose rate can be written as

(11) $$\dot{D}\;\left(t\right)=\dot{D}\;\left(t_{n-1}\right)+\frac{\dot{D}\left(t_{n}\right)-\dot{D}\left(t_{n-1}\right)}{t_{n}-t_{n-1}}\left(t-t_{n-1}\right)$$

where *t_n_* is the time of the *n^th^* scan. If the biological clearance rate *λ_1_* can be estimated from the last two or three measurements, we can write the absorbed dose rate function as

$$\dot{D}\;\left(t\right)=\dot{D}\;\left(t_{x}\right)e^{-\lambda_{1}\left(t-t_{x}\right)}\cdot e^{-\lambda \left(t-t_{x}\right)}.$$

Combining the exponentials yields:

(12) $$\dot{D}\;\left(t\right)=\dot{D}\;\left(t_{x}\right)e^{-\lambda_{e}\left(t-t_{x}\right)},$$

where similarly as before $\lambda_{e}=\lambda_{1}+\lambda$. For example, if we have four measurements and *λ_1_* is obtained by considering the final two measurements, then the absorbed dose rate function will be piecewise linear up until *t_x_ *= *t*_3_, then exponentially decaying for *t > t_x_*. In this case it is not necessary to resort to linear interpolation between the final two measurements. It is worth noting that it may be possible for the final measurement to be larger than the one preceding it, due to the uncertainties in quantitating activity with PET or SPECT imaging, such as in the case of high RPT retention in a tumor. In these cases, *λ_1_ *= 0 is often be assumed, implying unchanging tracer concentration. With this assumption, the absorbed dose rate will still decrease, as dictated by physical decay.

Defining the absorbed dose rate function in such a piecewise linear‐exponential way is consistent with ICRU guidelines.^6^, ^7^ Therefore, to remain consistent with existing dosimetry best practice, we will follow this approach when calculating both the total absorbed dose and the *G* factor.

Having defined the absorbed dose rate function, we will now evaluate *R*. A purely analytical solution is possible, however, calculating *G* for a linearly increasing absorbed dose rate would involve integrating products of polynomials and exponentials, which would result in complex and poorly generalizable closed‐form expressions. It is more generalizable to instead perform the integration numerically, as described by Hobbs and Sgouros in 2009.^21^ They proposed to use a dynamic programming algorithm combined with trapezoid double integration to calculate R. Their approach involves splitting the integral range into m bins of length dt, and calculating the inner integral, *M*, recursively

$$\begin{array}{cc} M\;\left(m\right) & =M\left(m-1\right)+\frac{1}{2}\left(\dot{D}\left(m\right)e^{\mu t\left(m\right)}\right.\\ & \left.+\dot{D}\left(m-1\right)e^{\mu t\left(m-1\right)}\right)dt, \end{array}$$

then the outer integral, *N*, recursively

$$\begin{array}{cc} N\;\left(m\right) & =N\left(m-1\right)+\frac{1}{2}\left(\dot{D}\left(m\right)M\left(m\right)e^{-\mu t\left(m\right)}\right.\\ & \left.+\dot{D}\left(m-1\right)M\left(m-1\right)e^{-\mu t\left(m-1\right)}\right)dt, \end{array}$$

such that

$$G=\frac{N\left(max\;time\;bin\right)}{D^{2}/2}\;.$$

While their method is completely general, it does involve calculating an exponentially increasing term with a growth factor *μ*, which has the potential to cause overflow errors if the convergence to the true solution is too slow, such as the in the case of slow RPT clearance when the time must be extrapolated considerably. For example, if the repair half‐time is relatively low, around 30 min, an interim calculation could result in values over 10^100^ after just 7 days, or around one ^177^Lu half‐life. Specifically, this problem becomes evident during the calculating of *M*(m), which diverges if μt(m) is large. In light of these limitations, we adopt the method of Hobbs and Sgouros in our calculations until *t_x_* but opt for solving the integral analytically once we start extrapolating.

We will now derive an expression for the *G* factor using our hybrid numerical‐analytical approach, assuming a single‐phase exponential repair model. Starting from *R* as defined in Equation 4, we first split the integrals into two parts at *t_x_*, separating the numerical and analytical contributions:

$$\begin{array}{cc} R & =\int_{0}^{t_{x}}dt\;\dot{D}\left(t\right)\int_{0}^{t}dt^{\prime }\dot{D}\left(t^{\prime }\right)e^{-\mu \left(t-t^{\prime }\right)}+\int_{t_{x}}^{\infty }dt\;\dot{D}\left(t\right)\\ & \;\times \left(\int_{0}^{t_{x}}dt^{\prime }\dot{D}\left(t^{\prime }\right)e^{-\mu \left(t-t^{\prime }\right)}+\int_{t_{x}}^{t}d\tau \dot{D}\left(t^{\prime }\right)e^{-\mu \left(t-t^{\prime }\right)}\right). \end{array}$$

For *t < t_x_*, the calculation is purely numerical. Hence for simplicity, we rename the first double integral to *N* and the first inner integral of the second term to *M*, signifying the fact that these will be computed separately. Rewriting the above expression leads us to

$$R=N+\int_{t_{x}}^{\infty }dt\;\dot{D}\left(t\right)e^{-\mu t}\left(M+\int_{t_{x}}^{t}dt^{\prime }\dot{D}\left(t\right)e^{\mu t^{\prime }}\right).$$

For *t ≥ t_x_*, as described above, we extrapolate to infinity via a monoexponential characterized by $\lambda_{e}$. Plugging in the expression for absorbed dose rate in that regime yields:

$$\begin{array}{cc} R & =N+\int_{t_{x}}^{\infty }dt\;\dot{D}\left(t_{x}\right)e^{-\lambda_{e}\left(t-t_{x}\right)}e^{-\mu t}\\ & \;\times \left(M+\int_{t_{x}}^{t}dt^{\prime }\dot{D}\left(t_{x}\right)e^{-\lambda_{e}\left(t^{\prime }-t_{x}\right)}e^{\mu t^{\prime }}\right). \end{array}$$

After solving the integrals, *R* will take the following final form:

$$R=N+\frac{M\dot{D}\left(t_{x}\right)e^{-\mu t_{x}}}{\lambda_{e}+\mu }+\frac{{\dot{D}}^{2}\left(t_{x}\right)}{2\lambda_{e}\left(\lambda_{e}+\mu \right)}.$$

Intuitively, the first term relates to two unrepaired sublethal hits before *t*_x_, the second term corresponds to one unrepaired sublethal hit before *t*_x_ and one after *t*_x_, while the last term is related to two unrepaired sublethal hits after *t*_x_. It is worth noting that dividing the last term by ${\dot{D}}^{2}\left(t_{x}\right)/2\lambda_{e}^{2}$ produces the well‐known expression for the *G* factor, $\lambda_{e}/\left(\lambda_{e}+\mu \right)$ in the case of single‐phase exponential decay, as it should.^11^ A more detailed derivation is presented in the Appendix.

Therefore, the final *G* factor will take the following form:

(13) $$G=\frac{2}{D^{2}}\;\left(N+\frac{M\dot{D}\left(t_{x}\right)e^{-\mu t_{x}}}{\lambda_{e}+\mu }+\frac{{\dot{D}}^{2}\left(t_{x}\right)}{2\lambda_{e}\left(\lambda_{e}+\mu \right)}\;\right).$$

The RE in this case will be expressed as:

(14) $$\mathrm{RE}\;=1+\frac{2}{D\alpha /\beta }\left(N+\frac{M\dot{D}\left(t_{x}\right)e^{-\mu t_{x}}}{\lambda_{e}+\mu }+\frac{{\dot{D}}^{2}\left(t_{x}\right)}{2\lambda_{e}\left(\lambda_{e}+\mu \right)}\right).$$

As a sanity check, it can be shown through dimensional analysis that the units remain consistent.

### 2.2 Simulations of common absorbed dose‐rate scenarios

To validate the above expressions and their implementations, the resultant EQDX were compared using a set of simple simulated absorbed dose rate values at different times. Datapoints were defined to lie on either a biexponential or a monoexponential curve with predefined parameters or were arbitrarily defined to resemble a tumor with high radiopharmaceutical retention. Three or four datapoints were defined around time points commonly encountered in the clinic, namely after 1 h, 4 h, 24 h, and 72 h post‐administration. The numerical method of Hobbs and Sgouros was used as a baseline method and it was run until a set termination time, which was either 200 or 2000 h after injection. The equieffective dose in 2 Gy fractions (EQD2) was calculated for 1000 stopping times between 72 h and this maximum. The calculations were repeated for a set of repair half times, covering a range encompassing potentially feasible fast and slow repair rates.^13^

The computational time necessary for various calculation methods was also compared. Specifically, we implemented three separate approaches: symbolic double integration using Python's (version 3.11.5) SciPy package^22^ (version 1.11.1), the method of Hobbs and Sgouros, and our hybrid method. Convergence checks were included for the first two algorithms. Starting from 72 h, stopping times were incremented in 10h steps until the difference between subsequent computations was below 0.1%.

### 2.3 Clinical data

Patient data was downloaded from the deep blue network of the University of Michigan,^23^ which included four SPECT/CT activity maps of two patients each following a [^177^Lu]Lu‐DOTATATE injection, as well as segmentations for various organs and tumors at each time point. The activity maps were converted to absorbed dose maps by Hermes Voxel Dosimetry (version 1.1.0, Hermes Medical Solutions AB)^24^ assuming all scans were done 1 h after injection and assuming complete decay after each scan. The absorbed dose‐rate maps were generated by correcting for this 1 h decay correction and by multiplying the resultant absorbed dose map by the decay constant to undo the activity integration. The absorbed dose rate maps and binary segmentation data were imported into Python and the pixel data were converted to Numpy (version 1.24.3) arrays using PyDicom (version 2.2.2.).^25^, ^26^ The binary segmentation maps were multiplied voxel‐wise with the image voxeldata and the resultant non‐zero voxels were averaged to get volume‐level mean absorbed dose rates, which were used for further modeling and analysis. The models described above were implemented in Python. The necessary metadata, such as time of scan, was obtained from the DICOM header. Calculations using the fully numerical method were stopped right before overflow errors were encountered at 250 h post injection, when using T_rep_ = 0.5 h. We chose a fast repair time to highlight the potential issues the fully numerical method may produce.

## 3 RESULTS

### 3.1 Simulated measurements for validation of EQDX expressions

**FIGURE 1:**
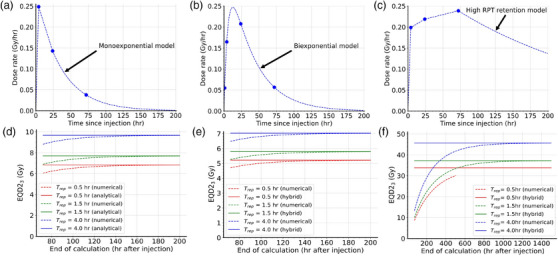
Simulation studies. Absorbed dose‐rates were defined by (a) biexponential, (b) monoexponential, or (c) high RPT retention models. Some clinically relevant measurement times and their corresponding absorbed dose rates are highlighted as single points. (d‐f) The validity of the analytical biexponential and the hybrid methods (solid lines) were compared against a fully numerical approach (dashed lines), for each model. The EQD2 was reported as a function of termination time for the numerical computations. Multiple repair half‐times are included. EQD2 equieffective dose in 2 Gy fractions; RPT, radiopharmaceutical therapy.

First, we validated our expressions using a simulated dataset with known underlying models, which are shown in Figure 1a‐c. These were selected to be representative of a range of clinically feasible tracer pharmacokinetic behaviors, with measurements sampled around typical post‐treatment scanning times after [^177^Lu]Lu‐DOTATATE injection. We compared the performance of the fully numerical method (dashed lines) with the analytical (Figure 1d) or hybrid (Figure 1e‐f) solutions (solid lines) as a function of stopping time for the numerical calculation.

The EQD2_3_ was found to depend on repair half‐time in each case, but the difference between the numerical and analytical or hybrid calculations were not. Figure 1d shows that the numerical method approaches the result obtained using Equation 10 if the calculation is extrapolated far enough. More specifically, a less than 2% difference in EQD2_3_ ($T_{rep}$ = 1.5h) is achieved if the calculation is stopped after 123 h in this particular case. Similarly, the numerical method approaches the result obtained using Equation 14, as shown in Figure 1e‐f. The minimum stopping time to achieve the same difference is 120 h when the simulated datapoints were lying on a monoexponential decay curve, and 890 h when the data points simulated high tracer retention. In fact, the numerical method failed to compute in the latter case due to overflow errors during interim calculations, and only when the repair half‐time was at least 1.5 h could it reach such an accuracy. The high required extrapolation time in Figure 1f is due to physical decay being assumed to be the only source of absorbed dose rate loss.

We benchmarked the time to compute both the hybrid and fully numerical methods against symbolic double integration, a possible alternative also not susceptible to overflow errors. The results are shown in Table 1.

**TABLE 1:** The time to reach a stable numerical result is shown for three models, calculated using three different approaches.

	Monoexponential model	Biexponential model	High RPT retention model
Hybrid method (ours)	**0.008 ± 0.002**	**0.008 ± 0.003**	**0.013 ± 0.008**
Fully numerical method	0.10 ± 0.01	0.12 ± 0.01	0.6 ± 0.1
Symbolic double integration	60 ± 5	138 ± 2	450 ± 10

*Note*: Calculations were repeated 10 times with α/β = 10 Gy and *T_rep_ *= 1.5 hr.Units are in seconds. The algorithms were implemented on an Intel(R) Xeon(R) W‐2235 CPU @ 3.80 GHz, using a single core. Fastest method in bold.

Abbreviation: RPT, radiopharmaceutical therapy.

The time to complete symbolic double integration was the slowest and most model‐dependent approach, on the order of min. The dynamic programming approach employed in the fully numerical method increased efficiency considerably, taking around half a second even for the high RPT retention model, where significant extrapolation was necessary. Our hybrid method improved computation times to around 10 ms, regardless of what model was analyzed.

### 3.2 The effects of simplifying dosimetry on EQD2

**FIGURE 2:**
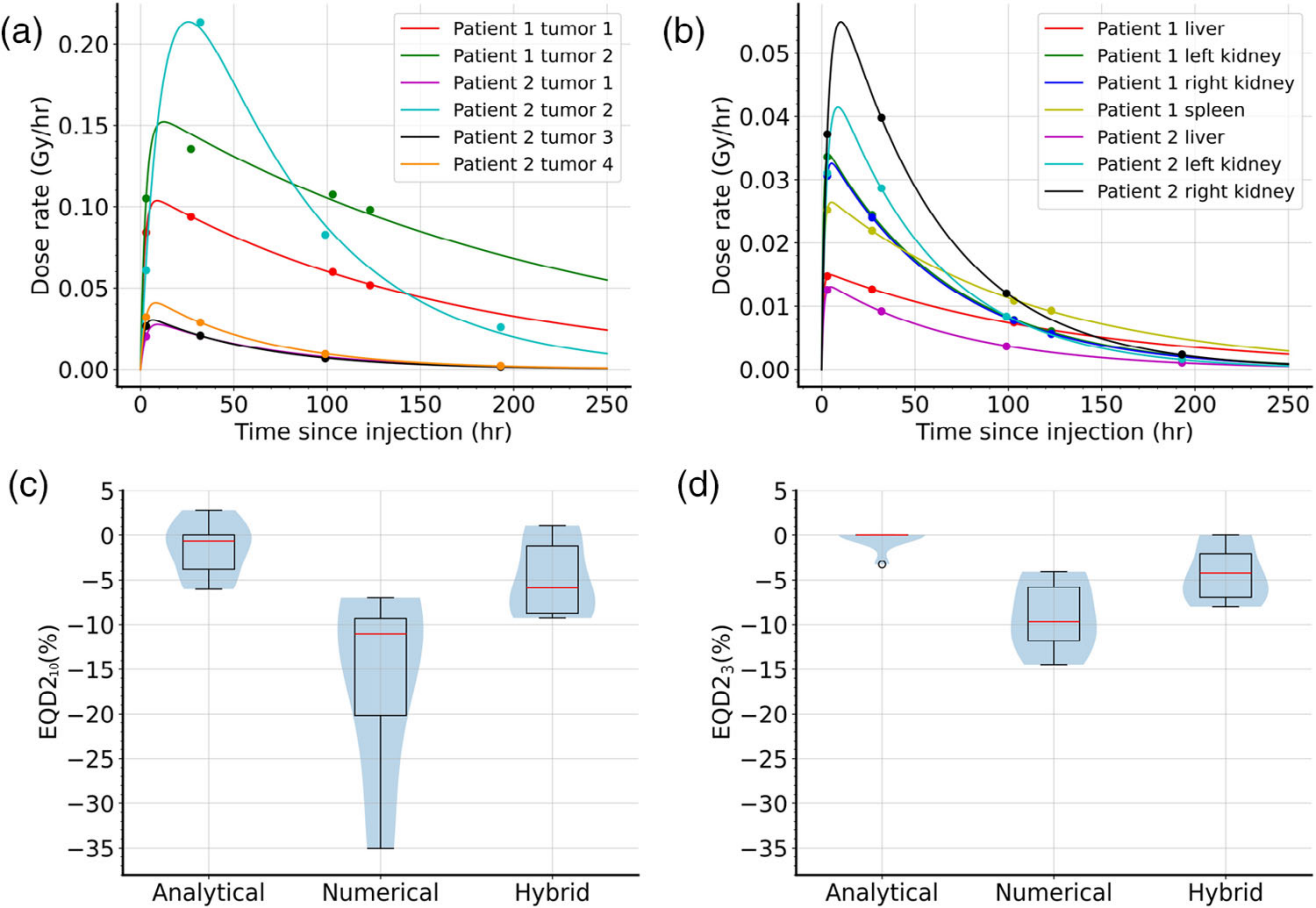
Clinical data and simplified dosimetry. The absorbed dose rate as a function of time since injection is shown for both patients, separately for (a) tumors and (b) organs. The measurements are shown as points and the solid lines represent the best fit biexponential function. The difference in EQD2_α/β_ is also shown for three different calculation methods when the last measurement is omitted, assuming α/β = 10 Gy for (c) tumors and α/β = 3 Gy for (d) organs and *T_rep_ *= 0.5 h for both. Overlaid box plots with outliers and violin plots visualize the distribution across different volumes of interest.

Once the expressions for the different methods and their implementations had been validated with simple models, we turned to compare their potential as a preferred method in a more realistic scenario with three measurement times. As discussed previously, the baseline model was defined to be a biexponential function fitted to four data points. Only the first three of these were used to compare the different methods. The absorbed dose rates were calculated on the voxel level and volume averages for a range of tumor and organ segmentations are shown in Figure 2a‐b, respectively. For most volumes, a biexponential function was an appropriate model, with tumor 2 of each patient exhibiting slight but non‐negligible deviations.

The relative differences in EQD2_α/β_ values compared to the four time point biexponential calculated by the analytical, numerical, and hybrid methods using only three time points are shown in Figure 2c‐d. The differences are largest using the numerical approach, due to insufficient extrapolation beyond 250 h caused by running into overflow errors, which is a fundamental consequence of this approach. In one instance, for example, the EQD2_10_ was underestimated by almost 35%, which equated to 6.7 Gy and 8.0 Gy, respectively. The analytical and hybrid methods performed similarly for tumors, with the latter performing slightly better in all but one instance, which was tumor 2 of patient 2. Specifically, the EQD2_10_ for tumors was underestimated on average by 15.6 ± 9.4%, 5.0 ± 4.2%, and 1.5 ± 2.9% using the numerical, hybrid, and analytical methods, respectively. For organs, the same methods yielded a negative difference of 9.1 ± 3.6%, 4.4 ± 3.3%, and 0.4 ± 1.1% in EQD2_3_.

## 4 DISCUSSION

### 4.1 Simulated measurements for validation of the expressions

In this article, we motivated and introduced two novel expressions for calculating the *G* factor, validated them, and investigated their accuracy when the dosimetry protocol is reduced from four to three scans. First, we solved Equation 4 and presented the solution in Equation 10. The assumptions that went into the derivation include complete decay of the radiation source and an initial absorbed dose rate of zero, which are representative for most radiopharmaceutical therapies. The presented equation is simple, which allows for the easy calculation of the EQDX when multi time‐point dosimetry is performed. We also presented Equation 14, which is a more relevant formulation when the biexponential model is inappropriate or there are too few measurements to estimate its parameters. We derived the expression assuming a piecewise‐linear absorbed dose rate function until a late time point, after which the signal was assumed to decay mono exponentially. This is consistent with the approach employed by several authors and software vendors when calculating the total absorbed dose. While Equation 14 requires numerical integration, the extrapolated portion is included analytically, bypassing the potential issues with short extrapolation times, convergence checks, and overflow errors. The former result is a more compact formulation of a more general expression that has already been derived elsewhere,^20^ while the latter is an extension of a fully numerical approach.^21^

Both new expressions were validated and compared against the fully numerical method introduced by Hobbs and Sgouros in 2009, in the case of simulated measurements from a purely biexponential and a purely monoexponential absorbed dose rate function, and also from measurements simulating high radiopharmaceutical retention in a tumor. As the fully numerical result depends on the time after injection when the calculation is terminated, we expected it to approach our values asymptotically, which was indeed what we observed. While we reported minimum stopping times for an accuracy of 2% in EQD2_3_, it must be noted that defining a single time at which a calculation should be terminated is difficult to define. Moreover, at sufficiently low absorbed dose rates repopulation may become significant, in which case more comprehensive radiobiological models should be used.^27^ For simplicity, we omitted regrowth from our models without loss of generality, as such factors are independent of *G* and RE, and hence do not affect our results.

It is important to note that the numerical method is also susceptible to overflow errors through an interim calculation involving a growing exponential in some cases when the RPT clearance is slow. When exactly the algorithm fails depends on several factors, the most important of which is the repair time constant *μ*. We found that in tumors with high retention and slow clearance, the numerical method fails well before it converges to the true value with any acceptable accuracy. We encounter such overflow errors when a calculation involves factors larger than what is possible to hold during floating point operations. It is worth noting that *μ* did not affect the accuracy of the methods otherwise.

Given the emerging importance of calculations on the voxel level, we sought to derive more scalable alternatives, both of which avoid issues during extrapolation. The increased efficiency of our method is quantified in Table 1, showing a 10‐50‐fold increase compared to the numerical method, and 4–5 orders of magnitude faster than symbolic double integration, reaching speeds of around 10 ms per calculation on a single CPU core on a consumer‐grade PC. Multithreading could push the efficiency even further, possibly generating full‐body EQDX maps in a matter of seconds—provided the radiobiological parameters are known.

### 4.2 The effects of simplifying dosimetry on the EQDX

Moving on from simulated data, we also sought to explore how a simplified dosimetry protocol affects radiobiological model accuracy. To our knowledge, this is the first such investigation, which is warranted as the medical physics community moves to recommend simplified dosimetry workflows. It is not immediately obvious that the lessons learnt from the accuracy of simplified dosimetry are directly translatable to the accuracy of the EQDX. Omitting the last measurement, we found that the analytical and hybrid methods performed well, while the numerical approach introduced large errors in multiple cases. Note that while some differences seem slight, it would be more accurate to multiply these by the number of fractions a patient is expected to undergo, which is usually four, at which point the differences become substantial. We also found that tumors are sometimes poorly described by a biexponential function or have slow clearance, which contributes to the errors seen in some cases with even the analytical solution. It is worth mentioning that the hybrid method performed similarly or better than the analytical approach in most cases, especially when the clearance was slow. Normal organ curves are well‐described by a biexponential model and hence the performance of the analytical method was excellent. The hybrid method underperformed in normal organs, presumably because the linear interpolation between measurements one and two cut off the peak of the baseline model, decreasing the total absorbed dose and hence the EQD2. This assumption can potentially explain why the differences are preferentially negative across all calculations. Such a problem is less of an issue for tumors with wider peaks and slow clearance, as there is a smaller chance of missing a pronounced peak. The inaccuracy of the numerical method can be attributed to the fact that the termination time was identically 250 h in all cases, which proved to be premature. This time was chosen such that overflow errors were just avoided, hence the calculation could not be run considerably longer, highlighting potential unavoidable problems using the fully numerical solution.

It is worth noting that for the liver lesions presented in this analysis, a higher dose per fraction EBRT schedule may be a more appropriate comparison, in line with recent advancements in stereotactic body radiotherapy.^28^ However, our results presented in Figure 2c‐d as relative percentages are independent of fraction size. This can be seen through Equations 1 and 2, as taking the ratio of two EQDX leads to a cancellation of the identical fraction‐size‐dependent denominators. Mathematically, using Equations 1 and 2 it can be shown that

$$\;\frac{EQDX_{\alpha /\beta }^{\left(1\right)}}{EQDX_{\alpha /\beta }^{\left(2\right)}}=\frac{EQD0_{\alpha /\beta }^{\left(1\right)}}{EQD0_{\alpha /\beta }^{\left(2\right)}}=\frac{BED_{\alpha /\beta }^{\left(1\right)}}{BED_{\alpha /\beta }^{\left(2\right)}}\;.$$

Moving forward, we recommend using the biexponential or hybrid models when calculating the EQD2, provided enough measurement times are available. The choice between them will need to be made depending on how the total absorbed dose is calculated, and depending on if the biexponential model is an appropriate model to describe the data. In which case this cannot be determined manually, such as on the voxel level, it is safer to opt for a data‐driven approach and implement the hybrid method. Our approach is expected to be most applicable for more comprehensive personalized dosimetry protocols, such as those found in clinical trials that seek to establish dose‐response models in RPT. However, our method is also compatible with simplified dosimetry schemes. For example, if patients undergo multiple scans after their first treatment fraction, that complete dataset can be used to establish personalized pharmacokinetic curves that are feasible inputs to our methods. Each subsequent fraction could use a single SPECT/CT scan if the tracer behavior is assumed to be identical and hence we can use the same parameters as inputs into our protocols, provided the fractions are sufficiently spaced apart in time.^18^

There are several limitations of the present analysis. While the expressions themselves have been validated, their starting assumptions may still carry errors. The LQ and EQDX models fared well in EBRT, but they have not been validated for RPT yet. It is often assumed that the α/β parameters derived from EBRT experiments are appropriate for RPT, which is not well‐founded and there is growing evidence to the contrary.^29^ Radiosensitivity parameters may not be appropriate, and the assumption that repair is described by a single‐phase exponential is not well‐founded. In vitro experiments and large‐scale clinical trial data are needed to confirm the validity of the LQ model. It is also not obvious that the repair function should take a monoexponential form and even if it does for EBRT, one must be cautious to extrapolate from conventional radiotherapy to other modalities. It is worth noting that other repair forms have been proposed, such as multiphase exponentials^30^ or a reciprocal form that is, the solution of a second‐order differential equation.^31^ In the latter case, none of the methods presented here apply, as the integral is not solvable analytically, which necessitates the use of symbolic double integration as the repair function could not be separated to simplify the calculation of the inner integral of Equation 4. Further limitations include the limited dataset we used here. More robust conclusions on the effects of simplification procedures can only be gauged by analyzing data from a larger cohort.

## 5 CONCLUSION

Our main aim was to derive and validate robust EQD*X* expressions that are relevant for absorbed dose rate functions commonly observed in RPT with [^177^Lu]Lu‐DOTATATE, and compare these against a fully numerical method. To that end, we introduced a simple closed‐form RE expression for a biexponentially decaying absorbed dose‐rate model and a partially analytical solution that is composed of a piecewise‐linear followed by an exponentially decaying absorbed dose‐rate function. Using simulated absorbed dose‐rate values, we found that the numerical method approached our results asymptotically the longer the computation was allowed to run, which confirms the validity of our expressions. Our methods were not dependent on a specific termination time and did not fail from overflow errors as was observed in the numerical method in some cases, while decreasing computation times significantly, paving the way for fast voxel‐level EQDX calculations.

We also investigated the effects of a simplified dosimetry workflow on radiobiological metrics calculated by using a fully numerical computation and our two expressions. We found that both new methods surpassed the numerical approach and provided closer estimates of the baseline model. We also found that using only three measurements, the RE expression for the biexponential absorbed dose rate function performed well for both organs and tumors, while the hybrid method performed slightly worse in organs and similarly in tumors. For tumors with slow clearance, the hybrid method outperformed both other approaches. The differences are non‐ negligible and potential numerical errors can negatively influence radiobiological model calculations, which could lead to incorrect dose limits, hampering clinical effectiveness, and adding to uncertainties associated with treatment outcome predictions.

The limitations of our proposed methods and analyses include the poor generalizability of our expressions to non‐exponential repair models, and the limited size of our dataset to draw conclusive results on the effects of simplifying the dosimetry workflow. We believe that our methods are suitable to be implemented alongside more complex dosimetry workflows, potentially providing more accurate estimates of the radiobiological effects of RPT.

## CONFLICT OF INTEREST STATEMENT

The authors declare no conflicts of interest.

## THE CASE OF NO REPAIR

The denominator in Equation 4 can easily be solved analytically:

$$\begin{array}{cc} \;R_{0} & =\int_{0}^{\infty }dt\;\dot{D}\left(t\right)\;\int_{0}^{t}dt^{\prime }\dot{D}\;\left(t^{\prime }\right)\\ & =\int_{0}^{\infty }dt\dot{D}\left(t\right)\;\left(D\left(t\right)-D\left(0\right)\right)\\ & =\int_{0}^{\infty }dt\dot{D}\left(t\right)D\;\left(t\right)=\frac{D^{2}}{2}\;\;\; \end{array}$$

if we made the assumption that *D*(0) = 0 and that τ *→ ∞*. These constraints assume that irradiation starts at the time of injection and that the radionuclide decays completely between fractions. The latter assumption is an approximation except for the last fraction, but the standard 8–10 week interfraction period encompasses 8.4‐10.5 ^177^Lu half‐lives, at which point physical decay reduces the decay rate to less than 0.5% of its original value. This is an upper limit, as we did not explicitly consider biological clearance. Therefore, assuming complete decay is a reasonable approximation, and we can let τ *→ ∞*.

## BIEXPONENTIAL ABSORBED DOSE RATE

We start from

$$R=\int_{0}^{\infty }dt\dot{D}\left(t\right)\int_{0}^{t}\;dt^{\prime }\dot{D}\left(t^{\prime }\right)e^{-\mu \left(t-t^{\prime }\right)}.$$

Substituting the absorbed dose rate function and factoring out *r*_0_ shows that the integral we need to solve is

$$\begin{array}{cc} & r_{0}^{2}\int_{0}^{\infty }dt\left(e^{-\lambda_{e_{1}}t}-e^{-\lambda_{e_{2}}t}\right)\int_{0}^{t}\;dt^{\prime }\\ & \;\left(e^{-\lambda_{e_{1}}t^{\prime }}-e^{-\lambda_{e_{2}}t^{\prime }}\right)e^{-\mu \left(t-t^{\prime }\right)}. \end{array}$$

Solving the inner integral gives

$$\begin{array}{cc} & r_{0}^{2}\int_{0}^{\infty }dt\left(e^{-\lambda_{e_{1}}t}-e^{-\lambda_{e_{2}}t}\right)\\ & \;\left(\frac{e^{-\lambda_{e_{2}}t}-e^{-\mu t}}{\lambda_{e_{2}}-\mu }-\frac{e^{-\lambda_{e_{1}}t}-e^{-\mu t}}{\lambda_{e_{1}}-\mu }\right). \end{array}$$

Expanding the integrand yields simple exponentials with various coefficients and exponents that can be integrated separately. Performing the integration gives a collection of constants, that after some algebra can be simplified to:

$$r_{0}^{2}\;\frac{{\left(\lambda_{e_{1}}-\lambda_{e_{2}}\right)}^{2}\left(\lambda_{e_{1}}+\lambda_{e_{2}}+\mu \right)}{2\lambda_{e_{1}}\lambda_{e_{2}}\left(\lambda_{e_{1}}+\lambda_{e_{2}}\right)\left(\lambda_{e_{1}}+\mu \right)\left(\lambda_{e_{2}}+\mu \right)}.$$

In order to calculate the *G* factor we must also compute *R*_0_, which will simply be

$$\;R_{0}=\frac{r_{0}^{2}}{2}\;{\left(\frac{1}{\lambda_{e_{1}}}-\frac{1}{\lambda_{e_{2}}}\right)}^{2}.$$

Dividing *R*_0_ by the previous expression recovers the *G* factor, after some simplification:

$$G=\frac{\lambda_{e_{1}}\lambda_{e_{2}}\left(\lambda_{e_{1}}+\lambda_{e_{2}}+\mu \right)}{\left(\lambda_{e_{1}}+\lambda_{e_{2}}\right)\left(\lambda_{e_{1}}+\mu \right)\left(\lambda_{e_{2}}+\mu \right)}\;.$$

Note that if the repair is fast, such that *μ ≈* 0, then *G *= 1, and we recover Equation 1, as we should in the case of complete repair of all sublethal damage and only one EBRT fraction. Conversely, if DNA repair pathways are impaired, then *μ → ∞* and *G *= 0, which will simplify Equation 3 to RE = 1. Intuitively, this will correspond to a situation when all damage is lethal, therefore the quadratic part of the LQ model vanishes.

## HYBRID METHOD

Starting from the following form:

$$\begin{array}{cc} R & =N+\int_{t_{x}}^{\infty }dt\;\dot{D}\left(t_{x}\right)e^{-\lambda_{e}\left(t-t_{x}\right)}e^{-\mu t}\\ & \;\times \left(M+\int_{t_{x}}^{t}dt^{\prime }\dot{D}\left(t_{x}\right)e^{-\lambda_{e}\left(t^{\prime }-t_{x}\right)}e^{\mu t^{\prime }}\right). \end{array}$$

we first expand the brackets, such that

$$\begin{array}{cc} & N+M\dot{D}\left(t_{x}\right)e^{\lambda_{e}t_{x}}\int_{t_{x}}^{\infty }dte^{-\left(\lambda_{e}+\mu \right)t}+{\dot{D}}^{2}\left(t_{x}\right)e^{2\lambda_{e}t_{x}}\\ & \;\times \int_{t_{x}}^{\infty }dte^{-\left(\lambda_{e}+\mu \right)t}\int_{t_{x}}^{t}dt^{\prime }e^{-\left(\lambda_{e}-\mu \right)t^{\prime }}, \end{array}$$

then we solve the integral of the second term and the inner integral of the third term. After moving constants outside of the integrals, we arrive at

$$\begin{array}{cc} & N+\frac{M\dot{D}\left(t_{x}\right)e^{-\mu t_{x}}}{\lambda_{e}+\mu }\\ & \;+\frac{{\dot{D}}^{2}\left(t_{x}\right)e^{2\lambda_{e}t_{x}}}{\lambda_{e}-\mu }\int_{t_{x}}^{\infty }dt\left(e^{-\left(\lambda_{e}+\mu \right)t}e^{-\left(\lambda_{e}-\mu \right)t_{x}}-e^{-2\lambda_{e}t}\right). \end{array}$$

Solving the last integral yields

$$N+\frac{M\dot{D}\left(t_{x}\right)e^{-\mu t_{x}}}{\lambda_{e}+\mu }+\frac{{\dot{D}}^{2}\left(t_{x}\right)e^{2\lambda_{e}t_{x}}}{\lambda_{e}-\mu }\left(\frac{e^{-2\lambda_{e}t_{x}}}{\lambda_{e}+\mu }-\frac{e^{-2\lambda_{e}t_{x}}}{2\lambda_{e}}\right),$$

which can be written as

$$N+\frac{M\dot{D}\left(t_{x}\right)e^{-\mu t_{x}}}{\lambda_{e}+\mu }+\frac{{\dot{D}}^{2}\left(t_{x}\right)}{\lambda_{e}-\mu }\left(\frac{1}{\lambda_{e}+\mu }-\frac{1}{2\lambda_{e}}\right),$$

that can be further simplified into its final form:

$$N+\frac{M\dot{D}\left(t_{x}\right)e^{-\mu t_{x}}}{\lambda_{e}+\mu }+\frac{{\dot{D}}^{2}\left(t_{x}\right)}{2\lambda_{e}\left(\lambda_{e}+\mu \right)}.$$

Note that the final *G* factor quoted in the main text can be recovered if the above expression is divided by the general equation derived for *R*_0_.
